# Fabrication of Bioinspired Hydrogels Using Carboxyphenylboronic Acid-Grafted Polyethylenimine and Polyvinyl Alcohol for Potential Wound Dressing Applications

**DOI:** 10.3390/biomimetics11070511

**Published:** 2026-07-21

**Authors:** Lei Nie, Zihan Sun, Shichang Cheng, Ling Wang

**Affiliations:** 1 College of Life Sciences, Xinyang Normal University, Xinyang 464000, China; 2 Dabie Mountain Laboratory of Henan Province, Xinyang 464000, China

**Keywords:** hydrogels, polyethylenimine, polyvinyl alcohol, bioadhesives

## Abstract

Tissue adhesives are gaining increasing attention as efficient alternatives to conventional wound closure methods, yet their clinical translation is often hindered by insufficient wet adhesion and inadequate biocompatibility. Drawing inspiration from nature’s robust wet-adhesion strategies, particularly dynamic covalent interactions and reversible crosslinking, we report a family of bioinspired composite hydrogels fabricated from 4-carboxyphenylboronic acid-grafted polyethylenimine (4-CPBA-PEI) and polyvinyl alcohol (PVA) that serve as versatile bioadhesives. The polyethylenimine with different molecular weights (18,000, 70,000, and 100,000 Da) was used to prepare the 4-CPBA-PEI derivatives via EDC/NHS-mediated amidation. The resulting hydrogels exhibited three-dimensional interconnected porous networks with tunable pore dimensions and equilibrium swelling ratios (ranging from 400% to 700%), closely correlated with the PEI molecular weight. Rheological measurements confirmed typical viscoelasticity, shear-thinning behavior, and outstanding self-healing performance, which are mainly attributed to hydrogen bonds and dynamic borate ester bonds in the network. The hydrogels firmly adhered to the surfaces of diverse matrices, such as glass, rubber, metal, plastic, wood, human skin, and wet mouse organs. Additionally, the obtained hydrogels exhibited high 2,2′-azino-bis (3-ethylbenzothiazoline-6-sulfonic acid) diammonium salt (ABTS) radical-scavenging activity (>85%), excellent hemocompatibility (hemolysis rate < 0.5%), and potent intracellular reactive oxygen species (ROS) scavenging activity. Cytocompatibility studies using NIH 3T3 fibroblasts demonstrated low cytotoxicity and favorable cytocompatibility. This biomimetic design yields multifunctional hydrogels that integrate tunable physical properties, wet-surface attachment, self-healing, antioxidant activity, and good biocompatibility, suggesting their potential as wound dressing candidates.

## 1. Introduction

Tissue adhesives have emerged as promising alternatives to traditional sutures and staples, which often cause secondary tissue damage, increase the risk of infection, require technical expertise, and are unsuitable for irregular or fragile tissues. An ideal bioadhesive should possess strong adhesion to moist biological surfaces, excellent biocompatibility, appropriate mechanical properties, and additional functions such as antibacterial activity, antioxidative capacity, and the ability to promote healing [1,2]. Hydrogel-based adhesives with hydrophilic three-dimensional (3D) networks have attracted great interest due to their unique ability to mimic the extracellular matrix (ECM), provide a moist wound environment, facilitate nutrient and oxygen exchange, and respond to physiological stimuli [3,4]. In recent years, the field of biomimetic materials has drawn significant inspiration from using nature’s ingenious strategies for achieving robust adhesion under challenging conditions [5,6]. Marine organisms such as mussels secrete protein-rich adhesive plaques containing 3,4-dihydroxyphenylalanine (DOPA), whose catechol groups can form reversible noncovalent interactions, such as metal coordination and π–π stacking, as well as hydrogen bonding [7,8]. These catechol-mediated interactions enable mussel-inspired hydrogels to dissipate mechanical energy and self-repair under external forces, which are highly desirable for wound dressings subjected to dynamic tissue movements. The above advantages have facilitated the development of biomimetic hydrogels with excellent adhesion properties for a wide range of biomedical applications.

Beyond catechol-based systems, the use of dynamic covalent bond connections has emerged as an effective biomimetic strategy for designing self-healing and stimuli-responsive hydrogels [9,10]. Phenylboronic acid (PBA) and its derivatives have received increasing attention in bioadhesive design owing to their unique ability to form reversible covalent bonds with *cis*-diol-containing molecules, such as sugars, glycoproteins, and polysaccharides present on tissue surfaces [11,12]. The reversible nature of boronate ester bonds, which can form and dissociate in response to changes in pH, glucose, and reactive oxygen species (ROS), mirrors the dynamic adaptability of biological systems [13,14]. This dynamic covalent chemistry enables reversible adhesion, which is particularly advantageous for applications involving tissue movement or repeated mechanical stress. Furthermore, the interaction between PBA and the hydroxyl groups of poly(vinyl alcohol) (PVA) provides a convenient crosslinking mechanism for hydrogel formation without external crosslinkers [15,16]. The reversibility of borate ester bonds also endows the hydrogels with self-healing capability, allowing them to spontaneously repair structural damage without compromising their integrity under deformation, a key feature inspired by the self-repair mechanisms observed in biological tissues [17]. Recent studies have demonstrated that PBA-functionalized hydrogels exhibit enhanced interfacial attachment, self-healing, and stimuli-responsive behavior, supporting their investigation as potential applications in wound dressings, drug delivery systems, and tissue engineering scaffolds [18,19].

PVA is a synthetic, water-soluble, non-toxic polymer, and it also displays excellent film-forming ability, chemical stability, and biocompatibility, which makes it a popular matrix for biomedical hydrogels [20]. However, pure PVA hydrogels suffer from low tissue adhesion and limited mechanical strength, which restrict their application as tissue-contacting or bioadhesive materials. To overcome these limitations, various functional groups and crosslinking strategies have been introduced. Polyethylenimine (PEI), a cationic polymer rich in primary and secondary amine groups, exhibits strong electrostatic interactions with negatively charged cell membranes and tissue surfaces, which may contribute to its adhesive properties [21,22]. Moreover, the high amine density of PEI has been reported to confer bacterial inhibition effects, though its cytotoxicity at high molecular weights and concentrations remains a major concern [23].

In this paper, we designed the composite hydrogels by combining 4-carboxyphenylboronic acid-grafted PEI (4-CPBA-PEI) with PVA, inspired by nature’s use of multiple complementary interaction mechanisms to achieve adaptable adhesion. This multi-interaction strategy is inspired by biological composite materials, which regulate their mechanical and adhesive performance through the hierarchical integration of diverse molecular interactions. Importantly, the molecular weight of PEI is expected to influence the extent of 4-CPBA introduction, crosslinking density, pore structure, and overall mechanical and biological performance. This structure-property relationship mirrors the way biological systems regulate material properties through precise molecular architectures. However, the PEI molecular weight influence on the properties of such PBA-PEI/PVA hydrogels has not been fully elucidated, and a comprehensive structure-property relationship is still lacking. Thus, 4-CPBA-PEI derivatives were synthesized using PEI with three different molecular weights (18,000, 70,000, and 100,000 Da), and the hydrogels were formed via dynamic borate ester crosslinking. We systematically characterized the hydrogels in terms of chemical structure, microstructure, swelling behavior, rheological properties, and self-healing ability. Their attachment behavior was qualitatively evaluated on various artificial surfaces and ex vivo mouse tissues. Furthermore, bacterial inhibition performance, antioxidant capacity, hemocompatibility, intracellular reactive oxygen species (ROS) fluorescence responses, cytocompatibility, and effects on cell migration were assessed to evaluate their potential for biomedical applications.

## 2. Materials and Methods

### 2.1. Chemicals

Polyethylenimine (PEI), 4-Carboxyphenylboronic acid (4-CPBA), 1-Ethyl-3-(3-dimethyllaminopropyl)carbodiimide hydrochloride (EDC), and N,N-dimethylformamide (DMF) were purchased from Macklin Co., Ltd. (Shanghai, China). Polyvinyl alcohol (PVA, Mw = 205,000, hydrolysis degree of 98%) and sodium hydroxide (NaOH) were obtained from Sinopharm Chemical Reagents Co., Ltd (Shanghai, China). N-hydroxysuccinimide (NHS) was purchased from Aladdin Co., Ltd. (Shanghai, China). Triton X-100 was obtained from Amresco Co., Ltd (Boise, USA). Millipore water was used as the ultrapure water, which was prepared from a Water System (Milli-Q50 SP, Millipore Corporation, Billerica, MA, USA). All chemical reagents were obtained from commercial suppliers and used without further purification.

### 2.2. Synthesis of 4-Carboxyphenylboronic Acid-Modified Polyethylenimine (4-CPBA-PEI)

The 4-CPBA-PEI polymer was synthesized using a previously reported approach with minor modifications [24]. Briefly, 1.0 g of PEI (PEI-1: 18,000 Da, PEI-7: 70,000 Da, and PEI-10: 100,000 Da) was dissolved in 20 mL MES (pH = 5.0) buffer under stirring at room temperature until complete dissolution. A total of 1.047 g of 4-CPBA was added to 17 mL of MES buffer (pH = 5.0). Then, 1.489 g of EDC and 0.8939 g of NHS (4-CPBA: EDC: NHS = 1:1.2:1.2, molar ratio) were added to the above solution, with 20 mL of DMF slowly added to promote dissolution. The mixture was stirred (500 rpm) at room temperature for 30 min to activate the carboxyl group. The activated 4-CPBA solution was mixed with the PEI-1, PEI-7, and PEI-10 solutions and stirred (500 rpm) at room temperature for 24 h. The reaction mixture was dialyzed in ultrapure Millipore water with packaging in a dialysis bag (molecular weight cutoff of 8000–14,000 Da); the water was changed every 8 h, and the dialysis process took 3 days. After dialysis and lyophilization, white flocculent CPBA-PEI-1, CPBA-PEI-7, and CPBA-PEI-10 polymers were obtained.

### 2.3. Fabrication of 4-CPBA-PEI/PVA Composite Hydrogels

Initially, a PVA solution (5 *w*/*v*%) was prepared by dissolving 5.0 g of PVA in 100 mL of Millipore water (90 °C in an oil bath, stirring at 500 rpm for 8 h). Subsequently, 0.0125 g of CPBA-PEI-1, CPBA-PEI-7, and CPBA-PEI-10 were added to a mixed solution of 470 μL Millipore water and 30 μL NaOH (5 wt%), yielding a 2.5 *w*/*v*% solution. The pH of the mixture before and after adding NaOH was 7.0 and 9.5, respectively. Then, 1 mL of PVA solution (5 wt%) was mixed with 0.5 mL of CPBA-PEI-1, CPBA-PEI-7, and CPBA-PEI-10 solutions individually, stirred for 3–4 s at room temperature, and hydrogel formation was observed, obtaining the CPA1, CPA7, and CPA10 hydrogels.

### 2.4. ^1^H Nuclear Magnetic Resonance (^1^H NMR) Spectroscopy

A total of 10 mg of 4-CPBA was dissolved in 0.6 mL of DMSO; 10 mg of PEI-1, PEI-7, and PEI-10 were dissolved in 0.6 mL of deuterated water (D_2_O) individually; and 10 mg of CPBA-PEI-1, CPBA-PEI-7, and CPBA-PEI-10 were dissolved in 0.6 mL of D_2_O and 25 μL of deuterated hydrochloric acid (DCI). The NMR tubes were used for subsequent tests, and the solution column height in the tube was adjusted to 4 cm. Next, a nuclear magnetic resonance spectrometer (JNM-ECZ600R/S3, JEOL, Tokyo, Japan) was employed to acquire the ^1^H NMR spectra of samples. According to the obtained ^1^H NMR spectra, the degree of substitution (SD) of phenylboronic acid groups on PEI was quantitatively calculated from the aromatic integration (6.5–8 ppm) relative to the methylene integration (2.5–3.5 ppm).

### 2.5. Fourier Transform Infrared Spectroscopy (FT-IR)

The data for 4-CPBA, PEI-1, PEI-7, PEI-10, CPBA-PEI-1, CPBA-PEI-7, CPBA-PEI-10, CPA1, CPA7, and CPA10 were recorded using a Fourier transform infrared spectrometer (FT-IR, Thermo Fisher, Nicolelis5, Waltham, MA, USA). During the FT-IR recording, the resolution was set at 1 cm^−1^, and the spectra in the range of 500–4000 cm^−1^ were acquired by accumulating 64 scans for each spectrum [25].

### 2.6. Scanning Electron Microscopy (SEM) Images

The tested wet hydrogels were treated by freezing (−20 °C for 24 h) and followed by lyophilizing for 3 days to obtain dry hydrogels. Next, the hydrogels were frozen in liquid nitrogen for 1 min, and the thin, uniform slices of dry hydrogels were prepared using a blade. After adhering the slice to the test stage with conductive adhesive, the surface was further treated via spraying platinum (120 s). The morphology and structure of the composite hydrogel were observed by a scanning electron microscope (Regulus8220, Tokyo, Japan), and the accelerating voltage was set at 30 kV [26]. Meanwhile, the pore size distribution of the hydrogel was measured by using ImageJ (Version 1.53).

### 2.7. Swelling Performance

A gravimetric approach was used to test the water absorption ability of hydrogels to evaluate their swelling performance. A total of 10 mg of the dried hydrogel (*m*_2_) was immersed in Millipore water at room temperature, and when the hydrogel reached the equilibrium swelling state in 36 h, the swollen mass (*m*_1_) was measured. The swelling ratio was calculated using the following Formula (1):

(1) $$\mathrm{Swelling}\;\mathrm{Ratio}\;(\%)\;=\;\frac{m_{1}\;-\;m_{2}}{m_{2}}\;\times \;100$$

### 2.8. Rheological Properties

The DHR-2 rheometer (TA, New Castle, DE, USA) was used to investigate the rheological properties of the CPA hydrogels. The storage modulus (G′) and loss modulus (G″) of hydrogels were recorded at physiological temperature (37 °C) in terms of time while using a steady strain at 1% and a frequency of 1 Hz. Furthermore, the amplitude sweep tests were accomplished using a fixed oscillation rate (1 Hz) in a strain range of 0.1–1000%. A parallel plate shape (diameter of 20 mm and interval of 1 mm) was used for all tests. Noted that the silicone oil was used to seal the edges to prevent water evaporation.

### 2.9. Self-Healing Performance

The self-healing properties of CPA hydrogels were investigated using macroscopic observation and rheological analysis. For the macroscopic observation, the wet hydrogels were separated into two pieces, using methylene blue to stain one of the pieces. Then, the stained and un-stained pieces recontacted along the incision interface. About 5 min later, the healing of the incisions was observed and photographed for documentation.

### 2.10. Qualitative Wet-Surface Attachment Test

The adhesive characteristics of CPA hydrogels were evaluated by contacting them with diverse surfaces, including glass, metal, plastic, rubber, wood, and skin. Their attachment to biological tissues was further examined using fresh ex vivo mouse tissues, including the liver, spleen, heart, lung, and stomach. The animal procedures were approved by the Ethics Committee of Xinyang Normal University (XFEC-2025-029). The attachment behavior of the hydrogels was photographed for documentation.

### 2.11. Bacterial Inhibition Test

The typical bacteria, including the Gram-negative *Escherichia coli* (*E. coli*, ATCC 25922) and the Gram-positive *Staphylococcus aureus* (*S. aureus*, ATCC 6538), were selected to investigate the bacterial inhibition of CPA hydrogels. Briefly, the bacterial suspensions (1 × 10^6^ CFU mL^−1^) were prepared for both bacteria. 10 mL of Luria–Bertani (LB) liquid medium was added to the sterile test tubes, and 10 μL of bacterial suspension was added as well. Next, 15 mg of lyophilized hydrogel was added to the above tubes as the experimental group. The control group did not have hydrogel. After incubating the mixture in a constant-temperature biochemical incubator (37 °C) using continuous shaking (180 rpm) for 6 h, the optical density at 600 nm values (OD_600_) of the solutions were measured using a microplate reader. The bacterial inhibition ratio was calculated according to the following Equation (2):

(2) $$\mathrm{Bacterial}\;\mathrm{Inhibition}\;\mathrm{Ratio}\;(\%)\;=\;\frac{k_{b}\;-\;k_{s}}{k_{b}}\;\times \;100$$

where *K_b_* indicates the absorbance of the control group, and *K_s_* indicates the absorbance of the sample group.

Subsequently, select the parallel and blank tubes with the best inhibitory effects from the samples. SEM images were employed to observe the morphology of bacteria.

### 2.12. Antioxidant Activity

The antioxidant activity of the CPA hydrogels was investigated using the 2,2′-azino-bis (3-ethylbenzothiazoline-6-sulfonic acid) diammonium salt (ABTS) radical scavenging assay. The ABTS radical cation (ABTS•+) stock solution (Macklin Co., Ltd., Shanghai, China) was prepared by mixing equal volumes of an aqueous ABTS solution (12 mg in 3 mL) and an aqueous potassium persulfate solution (2 mg in 3 mL). The mixture was incubated in the dark at 4 °C for 12 h. Before use, the resulting solution was diluted 23-fold with Millipore water to prepare the working solution. 20 mg of the dry samples were added to 5 mL of the ABTS•+ working solution and incubated in the dark at 30 °C with shaking at 150 rpm for 30 min. After incubation, the samples were allowed to settle, and the supernatant was collected. The absorbance of the supernatant was measured at 734 nm using a microplate reader. The ABTS scavenging activity was calculated according to the following Formula (3):

(3) $$\mathrm{ABTS}\;\mathrm{Scavenging}\;\mathrm{Activity}\;\left(\%\right)\;=\;\frac{A_{c\;}-\;A_{b}}{A_{c}}\;\times \;100$$

where A_c_ represents the absorbance values at 734 nm of the control group, and A_b_ indicates the absorbance values at 734 nm of the experimental groups.

### 2.13. Hemolysis Evaluation

First, the fresh mouse blood was added to normal saline (0.9 wt.%) to obtain the red blood cell suspension. Each dried hydrogel sample (2.5 mg) was placed in a 1.5 mL centrifuge tube containing 1 mL of 0.9% saline and preincubated at 37 °C for 30 min. Subsequently, 20 µL of red cell suspension was added to the centrifuge tube. For the negative control, 20 µL of red cell suspension was added to 1 mL of 0.9% saline. For the positive control, 20 µL of red cell suspension was added to 1 mL of ultrapure water. All groups were incubated at 37 °C for 1 h. After centrifuging (3000 rpm, 5 min), the supernatants were collected, and their absorbance was measured at 545 nm [27]. The hemolysis rate was calculated using the following Equation (4):

(4) $$Hemolysis\;Rate\;(\%)=\frac{A_{s\;}-\;A_{n}}{A_{p}\;-\;A_{n}}\;\times \;100$$

where *A_s_*, *A_p_*, and *A_n_* were the absorbance values corresponding to the sample group, the positive control group, and the negative control group, respectively.

### 2.14. Biocompatibility Evaluation

The biocompatibility of the hydrogel was evaluated using Cell Counting Kit-8 (CCK-8) analysis [28]. Following the ATCC instructions, NIH 3T3 fibroblasts (CRL-1658^TM^, ATCC, Manassas, WV, USA) were cultured in high-glucose Dulbecco’s Modified Eagle Medium (DMEM) supplemented with 10% fetal bovine serum (FBS) and 1% penicillin-streptomycin at 37 °C in a humidified 5% CO_2_ atmosphere. The cytotoxicity of CPA hydrogel was detected by using CCK-8. Lyophilized hydrogel samples (CPA1, CPA7, and CPA10) of equal mass were placed into centrifuge tubes and immersed in equal volumes of 75% ethanol for 12 h at room temperature. The samples were subsequently exposed to ultraviolet light (UV) for sterilization. After removal of the ethanol, the hydrogel samples were washed 3 to 5 times with PBS (pH = 7.4). Subsequently, an equal volume of complete culture medium was added to each sample, and the samples were incubated at 37 °C for 24 h to obtain the hydrogel extracts. NIH-3T3 cells grew well in 96-well plates containing the extracts, while the control group was inoculated with the cells into complete medium. After 1, 2, and 3 days of culture, 10 μL of CCK-8 reagent was added to each well, and the wells were incubated at 37 °C for 2 h in the dark. The resulting solutions were transferred to a new 96-well plate, and absorbance at 450 nm was measured with a microplate reader (SpectraMax 190, Molecular Devices, San Jose, CA, USA). Cell viability was calculated according to the following Equation (5):

(5) $$Cell\;viability\;(\%)=\frac{A_{s\;}-\;A_{b}}{A_{c}\;-\;A_{b}}\;\times \;100$$

where *A_s_*, *A_c_*, and *A_b_* refer to the absorbance values from the sample group, the control group, and the blank group, respectively.

The cytocompatibility of the CPA hydrogels was further evaluated using a Calcein acetoxymethyl ester/propidium iodide (Calcein-AM/PI, Biyuntian Biotechnology Co., Ltd., Shanghai, China) live/dead cell staining kit [29]. The cells were seeded in a 35 mm culture dish at a density of 5 × 10^5^ cells per well. The culture medium in the sample groups was then replaced with the corresponding hydrogel extracts prepared as described above, while cells cultured in complete medium were used as the control group. After incubation for 24 h, the cells were stained with Calcein-AM and PI according to the protocols. Fluorescence images were subsequently obtained using a fluorescence microscope (Nikon, Tokyo, Japan).

### 2.15. Cell Scratch Assay

The cell scratch assay was used to observe the effect of CPA hydrogel on cell migration. Place the scratch insert (Culture-Insert 3 Well in µ-Dish 35 mm, ibidi GmbH, Martinsried, Germany) on a six-well plate. After that, NIH 3T3 cells were inoculated into the plate (cell density: 5 × 10^4^ cells per well), and the scratch insert was removed after 24 h of cultivation. Then, the plate was washed 3 times using PBS. Next, the fresh hydrogel extract (1 mL) was added as the sample group, and the fresh culture medium (1 mL) was added as the control group. The photos after culturing at 0 h, 6 h, and 12 h were taken to investigate the scratch widths [30]. In addition, the cell migration rate was statistically analyzed using ImageJ (Version 1.53). The calculation for Formula (6) is shown as follows:

(6) $$\mathrm{Migration}\;\mathrm{Rate}\;\left(\%\right)=\frac{S_{0}\;-\;S_{n}}{S_{n}}\;\times \;100$$

where *S*_0_ represents the initial scratch width, and *S_n_* represents the width of the scratch at 6 h and 12 h.

### 2.16. Statistical Analysis

Three independent replicates were used for all experiments, and the data were presented as the mean ± standard deviation (SD). The statistical analysis was accomplished using one-way ANOVA and two-way ANOVA (GraphPad Prism 10.0 software, San Diego, CA, USA) of the hemolysis assay. CCK-8 data were analyzed using two-way ANOVA (GraphPad Prism 10.0 software, San Diego, CA, USA). All data were analyzed by one-way ANOVA using SPSS 28.0 software (Chicago, IL, USA) with Tukey’s multiple comparisons test for all post hoc analyses. Significant differences are displayed as * *p* < 0.05, ** *p* < 0.01, *** *p* < 0.001, and **** *p* < 0.0001. “ns” means no significant difference.

## 3. Result and Discussion

### 3.1. Fabrication of CPA Hydrogels

**Figure 1:**
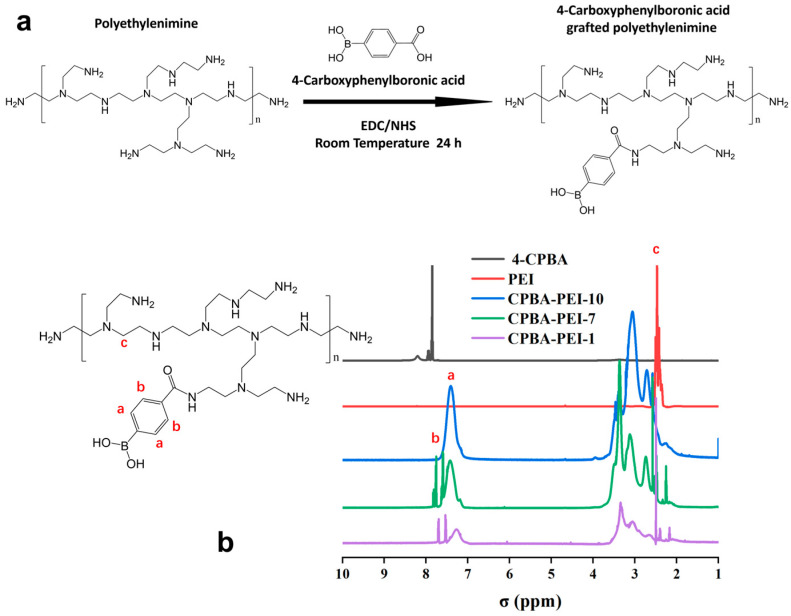
(**a**) CPBA-PEI was synthesized through an EDC/NHS-mediated amidation reaction. (**b**) ^1^H NMR spectra of 4-CPBA, PEI, CPBA-PEI-1, CPBA-PEI-7, and CPBA-PEI-10 in D_2_O.

A series of 4-CPBA-PEI derivatives with varying degrees of grafting was synthesized via EDC/NHS-mediated amidation, in which 4-CPBA was covalently conjugated to the backbone of branched PEI. The synthetic pathway is depicted in Figure 1a. Stable amide linkages were formed between the carboxyl groups of 4-CPBA and primary/secondary amines on PEI side chains. The resulting polymers retained the intrinsic cationic features of PEI while incorporating phenylboronic acid moieties, thereby paving the way for the fabrication of PBA-containing functional biomaterials. To verify the reaction and analyze the structure of the products, ^1^H NMR characterization was performed on 4-CPBA, different molecular weights of PEI, and the prepared 4-CPBA-PEI (CPBA-PEI-1, CPBA-PEI-7, and CPBA-PEI-10). The results are shown in Figure 1b. In the ^1^H NMR spectrum of PEI, the strong signal at 2.0 to 3.0 ppm corresponds to the methylene protons (-CH_2_-) in different chemical environments within the PEI backbone (peak c), a characteristic signal of PEI. For 4-CPBA, typical aromatic proton signals were observed in the range of 7.5–8.0 ppm. The resonance near 7.8 ppm corresponded to aromatic protons adjacent to the carboxyl group (peak b), while the signal around 7.5 ppm was attributed to the aromatic protons next to the boronic acid group (peak a). All modified 4-CPBA-PEI samples exhibited both methylene proton signals of the PEI backbone (2.0–3.0 ppm) and aromatic proton signals of 4-CPBA (7.0–8.0 ppm). The coexistence of these characteristic signals provides qualitative evidence for the successful conjugation of 4-CPBA onto PEI chains.

Further spectral analysis revealed that the relative intensity of aromatic proton signals increased gradually with increasing molecular weight of PEI, indicating an increased number of grafted phenylboronic acid groups. According to quantitative calculations of the substitution degree (SD) of phenylboronic acid groups on PEI, the SD for CPBA-PEI-1, CPBA-PEI-7, and CPBA-PEI-10 was 18.38 ± 1.01%, 22.19 ± 1.21%, and 36.42 ± 1.35%, respectively. Collectively, 4-CPBA-PEI derivatives with different grafting rates were successfully prepared. Specifically, CPBA-PEI-10 exhibited the highest intensity of aromatic proton signals and thus the highest degree of grafting, whereas CPBA-PEI-1 showed the weakest aromatic hydrogen peaks and the lowest degree of grafting.

**Figure 2:**
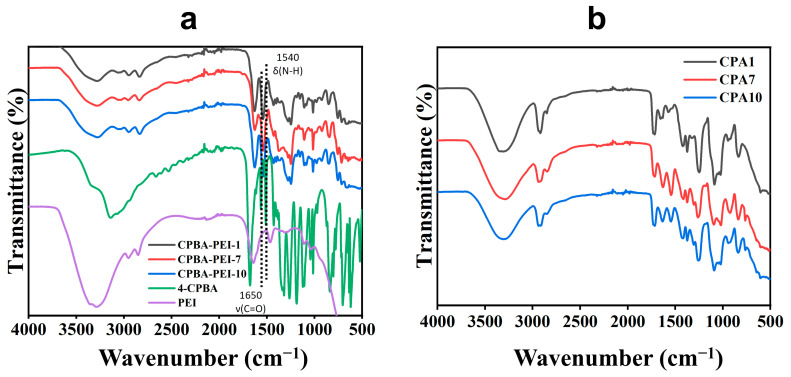
(**a**) FT-IR spectra of 4-CPBA, PEI, CPBA-PEI-1, CPBA-PEI-7, and CPBA-PEI-10. (**b**) FT-IR spectra of CPA1, CPA7, and CPA10.

Fourier transform infrared (FTIR) spectroscopy was performed to characterize neat PEI, 4-CPBA, and 4-CPBA-PEI derivatives prepared using PEI with different molecular weights, and the results are presented in Figure 2a. In contrast, all 4-CPBA-PEI samples exhibited characteristic absorption peaks of amide bonds. The band at approximately 1650 cm^−1^ was assigned to the amide I band, corresponding mainly to the stretching vibration of C=O in the amide linkage, while the absorption at 1540 cm^−1^ was assigned to the amide II band, originating from the bending vibration of N-H in the amide linkage. The appearance of these new peaks directly confirmed the successful formation of amide bonds between the carboxyl groups of 4-CPBA and the amino groups of PEI, verifying the covalent conjugation of phenylboronic acid moieties onto PEI. Further comparison of 4-CPBA-PEI samples prepared using PEI with different molecular weights (Figure 2b) revealed that the relative intensities of the characteristic amide peaks at 1650 cm^−1^ and 1540 cm^−1^ varied with PEI molecular weight, indicating a significant influence of PEI molecular weight on the grafting efficiency of phenylboronic acid moieties under identical reaction conditions. These results are consistent with the ^1^H NMR characterization and further confirm the successful preparation of 4-CPBA-PEI derivatives based on PEI with different molecular weights.

### 3.2. Microstructure and Water Absorption Properties of CPA Hydrogels

**Figure 3:**
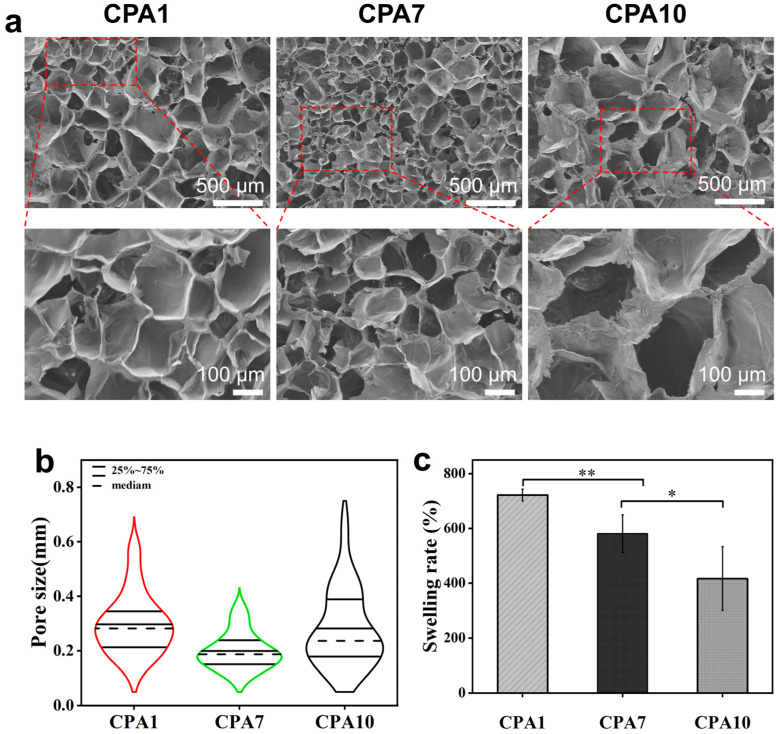
Micro-morphological and swelling properties assessment of CPA hydrogels. (**a**) SEM images of CPA hydrogels. (**b**) The pore size distribution of CPA hydrogels was statistically analyzed in ImageJ using five SEM images. (**c**) Swelling ratios of CPA hydrogels tested in Millipore water (n = 3), * *p* < 0.05, and ** *p* < 0.01.

The physicochemical properties of 4-CPBA-PEI/PVA hydrogels were investigated, including microstructure, swelling capability, rheological characteristics, self-healing ability, and wet-surface attachment behavior. The morphological characteristics of freeze-dried CPA1, CPA7, and CPA10 were observed by SEM (Figure 3a). All CPA hydrogels exhibited a porous and interconnected three-dimensional network structure, which may facilitate fluid absorption and mass exchange and is a desirable characteristic for hydrogel wound dressing materials. CPA1 exhibited a relatively dense, heterogeneous morphology with well-defined pore walls. CPA7, in contrast, featured a more uniform and regular porous network with slightly smaller pore cavities. CPA10 displayed a heterogeneous structure with thicker pore walls and poorly defined boundaries, likely due to increased polymer chain entanglement at higher molecular weights.

Based on the SEM images, the pore sizes of the three hydrogels were statistically analyzed using ImageJ, as shown in Figure 3b. The lower-molecular-weight hydrogel (PEI 18,000 Da) exhibited larger pores, likely due to the formation of a less entangled polymer network. The medium molecular weight hydrogel (PEI 70,000 Da) exhibited a denser, more uniform structure with smaller pores. The high-molecular-weight hydrogel (PEI 100,000 Da) produced an intermediate pore size but a more heterogeneous architecture.

Figure 3c displays the equilibrium swelling ratios of CPA1, CPA7, and CPA10 hydrogels. Notably, the swelling ratio varied inversely with molecular weight: CPA1 exhibited the highest, reaching 700%, followed by CPA7 at approximately 580%, and CPA10 the lowest at 400%. CPA1, which had the largest pore size, also exhibited the strongest water-uptake capacity. However, although CPA7 exhibited a smaller pore size than CPA10, it showed a relatively higher swelling ratio, indicating that factors other than pore size, such as polymer hydrophilicity, crosslinking density, and network flexibility, may also play important roles in determining the swelling capacity of the hydrogels. These results suggest that the molecular weight of PEI influences both the pore size and the swelling ability of the hydrogels, but not directly in a proportional manner. The relatively high swelling properties of the CPA hydrogel may be beneficial for absorbing wound exudate when used as a wound dressing [31].

### 3.3. Rheological Behavior and Self-Healing Ability of CPA Hydrogels

**Figure 4:**
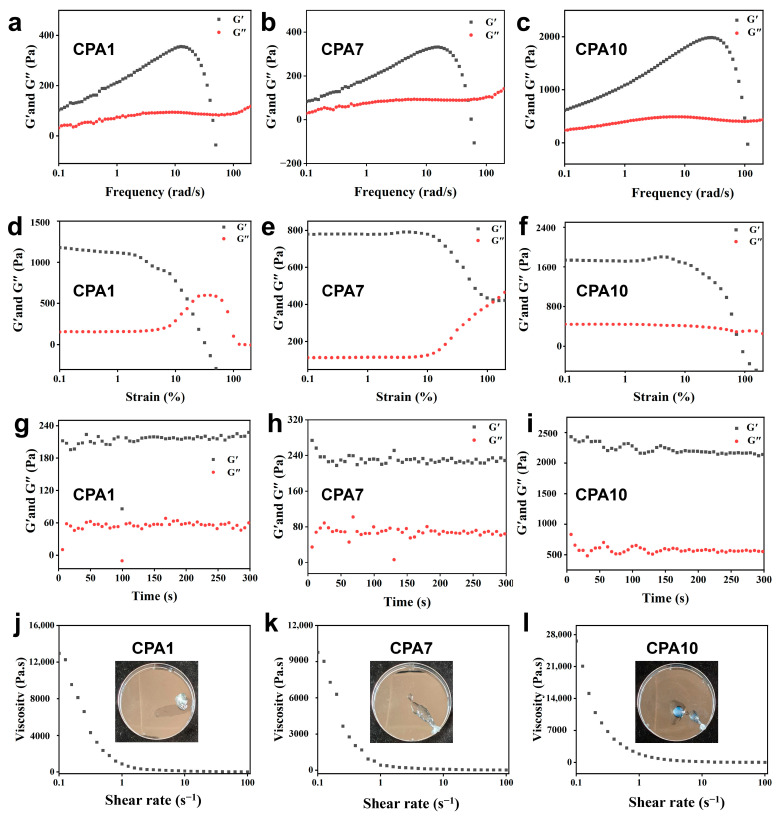
The storage modulus (G′) and loss modulus (G″) of CPA hydrogels in terms of (**a**–**c**) frequency, (**d**–**f**) strain, and (**g**–**i**) time. (**j**–**l**) The rheological behavior of CPA hydrogels.

The rheological behavior of the fabricated CPA hydrogel was evaluated using a rheometer to investigate the viscoelastic properties and potential processability. The storage modulus (G′) and loss modulus (G″) were recorded in terms of frequency, strain, and time using a rheometer (Figure 4a–i). For all CPA hydrogels, G′ values were apparently larger than G″ values in terms of frequency and time, revealing their typical rheological behavior. With the increase in frequency and strain, G’ values sharply decreased and intercrossed with the increased G″ values, indicating the collapse of CPA hydrogels. As shown in Figure 4j–l, the apparent viscosity of all hydrogel formulations dropped sharply as the shear rate increased from 0.1 to 100 s^−1^, indicating typical non-Newtonian pseudoplastic behavior. Such shear-thinning characteristics are essential for minimally invasive delivery and in situ gelation.

**Figure 5:**
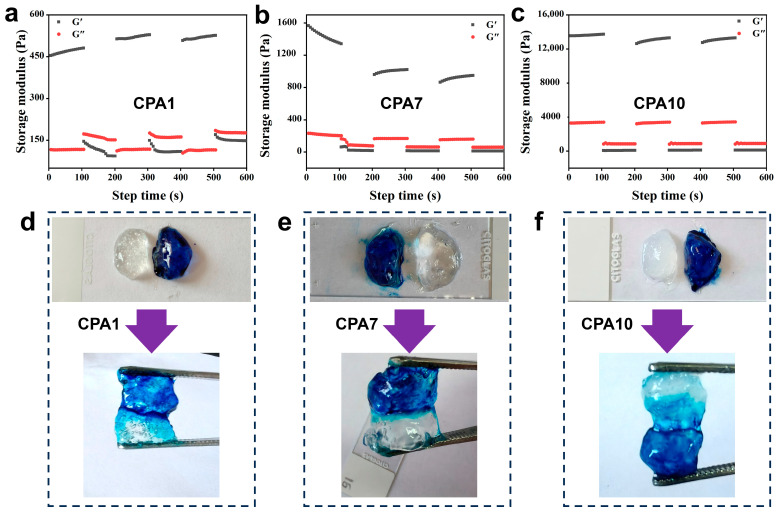
The self-healing capability of (**a**) CPA1, (**b**) CPA7, and (**c**) CPA10 hydrogels was evaluated using 3-cycle step strain tests. (**d**–**f**) The photos showed the self-healing behavior of CPA hydrogels.

Furthermore, the self-healing behavior of CPA hydrogels was investigated by step-strain rheological tests (Figure 5a–c) and macroscopic observation (Figure 5d–f). Samples CPA1, CPA7, and CPA10 hydrogels all displayed self-healing ability. The separated parts could be reconnected within 5 min, and the reconnected samples remained intact. The self-healing performance of CPA hydrogel stems from the synergistic effect of dynamic borate ester bonds and hydrogen bonds. The dynamic borate ester bonds facilitate network reconstruction, while the hydrogen-bonding interactions may further contribute to the recovery of network integrity [17,32,33].

### 3.4. Adhesive Properties of CPA Hydrogels

**Figure 6:**
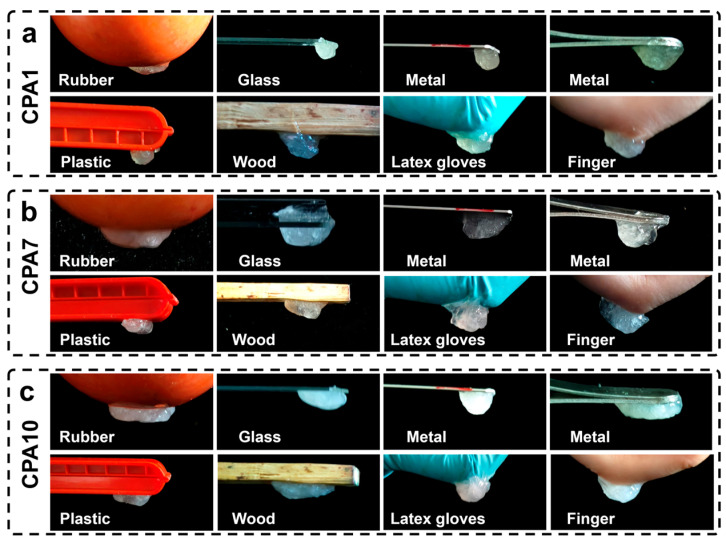
Photos showed CPA hydrogels could firmly adhere to the surface of different materials, including rubber, glass, metal, plastic, wood, latex gloves, and human skin. (**a**) CPA1, (**b**) CPA7, and (**c**) CPA10.

**Figure 7:**
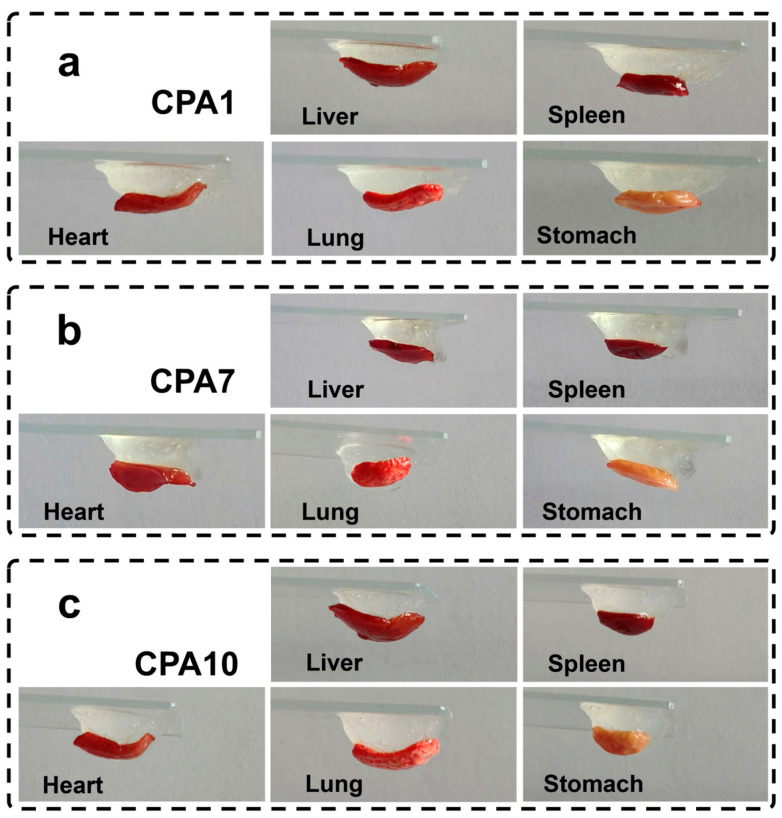
Different organs, such as the liver, spleen, heart, lung, and stomach, were used to further evaluate the adhesive strength of CPA hydrogels. (**a**) CPA1, (**b**) CPA7, and (**c**) CPA10.

The adhesive properties of CPA hydrogels were qualitatively evaluated using macroscopic attachment tests (Figure 6 and Figure 7). CPA hydrogels could firmly adhere to the surface of different substrates, such as rubber, glass, metal, plastic, wood, latex gloves, and human skin (Figure 6a–c). In addition, the CPA hydrogels demonstrated visible attachment to several organs, including liver, spleen, heart, lung, and stomach (Figure 7a–c). The wet-surface attachment of this hydrogel may stem from dynamic borate ester bonds formed between phenylboronic acid and hydroxyl/diol moieties on the substrate surface, together with synergistic hydrogen-bonding interactions between amino and hydroxyl groups [34,35]. These qualitative observations suggest that CPA hydrogels possess wet-surface attachment capability, which may be beneficial for maintaining close contact with tissue surfaces when used as wound dressing materials.

### 3.5. Bacterial Inhibition Performance of CPA Hydrogels

**Figure 8:**
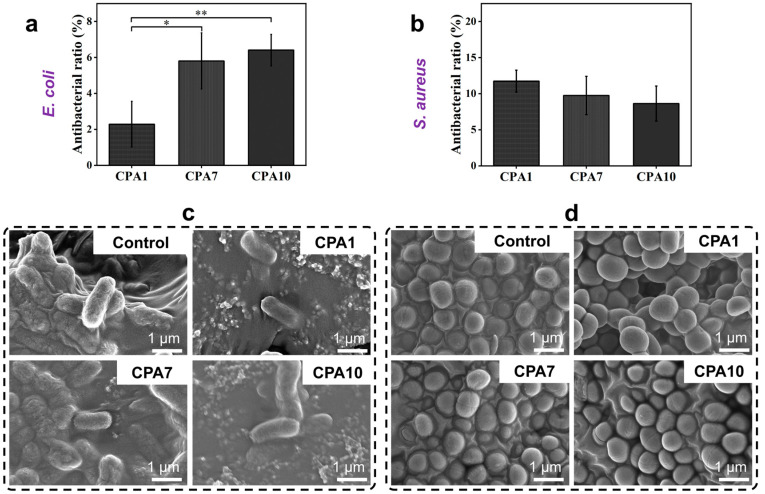
The bacterial inhibition activities of the fabricated hydrogels were evaluated using *S. aureus* and *E. coli*. The bacterial inhibition ratios of CPA hydrogels against *E. coli* (**a**) and *S. aureus* (**b**) for 6 h. SEM images of *E. coli* (**c**) and *S. aureus* (**d**) after incubating with CPA hydrogels for 6 h, * *p* < 0.05, and ** *p* < 0.01.

Using typical Gram-negative *E. coli* and Gram-positive *S. aureus* as model bacteria, the bacterial inhibition performance of the developed hydrogel was systematically evaluated through liquid culture inhibition assays and agar plate colony counting analysis (Figure 8a–d). For *E. coli*, the bacterial inhibition rate increased with increasing PEI molecular weight, and CPA10 exhibited the highest inhibitory effect among the tested groups, with significant statistical differences observed in comparisons (* *p* < 0.05, ** *p* < 0.01). By contrast, the inhibitory effect against *S. aureus* showed only a slight overall increase, with no significant differences among the groups. However, the overall bacterial inhibition rates of CPA hydrogels were below 15%, indicating that the antibacterial effect of the hydrogel system was limited under the experimental conditions used. Furthermore, after incubating the bacteria with the hydrogels for 6 h, their morphology was examined using a scanning electron microscope, as shown in Figure 8c,d. It can be observed that after incubation with the CPA hydrogel, the numbers of *E. coli* and *S. aureus* decreased to some extent, but there was no significant change in bacterial structure. These results indicate that the CPA hydrogels exhibited limited antibacterial effects under the present experimental conditions.

It is well known that the antibacterial activity of PEI is strongly dependent on its positive charge density, which is primarily contributed by protonatable primary amine groups (–NH_2_) [36]. Nevertheless, during the synthesis of CPA hydrogels, the primary amine groups of PEI are consumed via chemical modification. This may explain the relatively weak antibacterial performance of CPA hydrogels. Secondly, upon cross-linking of PEI and PVA to form a hydrogel network, dense intermolecular hydrogen bonds are generated, which may reduce the effective positive charge density on the hydrogel surface, thereby impeding the initiation of the cationic contact-killing effect [37].

### 3.6. Antioxidant, Hemocompatibility, and Intracellular ROS Level Investigation of CPA Hydrogels

**Figure 9:**
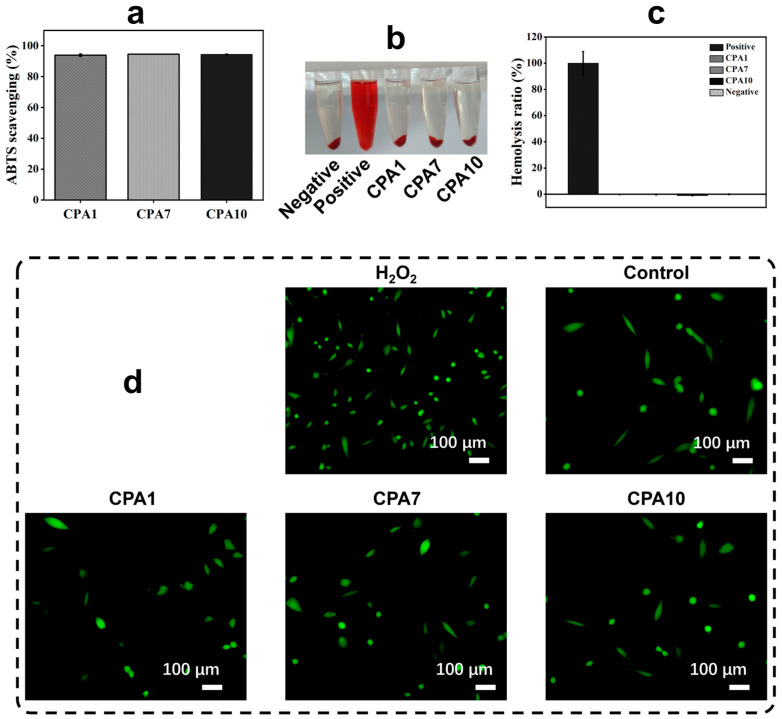
(**a**) ABTS radical scavenging rate of CPA hydrogels, which was tested for 30 min. (**b**) The hemolysis assay was photographically recorded. (**c**) Hemolysis ratios. (**d**) The intracellular ROS levels were evaluated in NIH 3T3 cells, in which the cells were pre-treated with H_2_O_2,_ followed by DCFH-DA staining.

As a critical inducer of membrane lipid peroxidation, protein denaturation, and DNA damage, oxidative stress plays a vital role in the development of inflammation, senescence, and diverse degenerative disorders [38,39]. The antioxidant activity of CPA hydrogels was investigated via the ABTS radical scavenging assay (Figure 9a). The ABTS scavenging ratios of all CPA hydrogels were over 85%, and there were no significant differences among CPA1, CPA7, and CPA10 hydrogels. The observed radical scavenging performance may be associated with the abundant amine groups (-NH_2_, -NH-) and phenylboronic acid-containing moieties within the hydrogel network. The primary and secondary amine groups of PEI chains may serve as hydrogen- or electron-donating sites [40], while the grafted 4-CPBA groups may interact with oxidative species, resulting in a synergistic radical-scavenging effect [41]. Furthermore, the hydrophilic network formed by PVA and 4-CPBA-PEI may facilitate the contact between the water-soluble free radicals and the available reactive groups within the hydrogels. The similar scavenging ratios of CPA1, CPA7, and CPA10 suggest that variation in PEI molecular weight did not substantially affect their ABTS radical scavenging performance under the present experimental conditions [42].

Given the potential biomedical applications of CPA hydrogels, their hemolytic effects were tested using mouse blood. From the photos after the testing, the supernatants of the CPA hydrogels and the negative group were transparent, with no obvious red coloration indicating hemoglobin release (Figure 9b). The hemolysis rate of all CPA hydrogels was almost 0%, which was consistent with that of the negative group (Figure 9c), indicating low hemolytic potential under the present experimental conditions. The obtained hemolysis rates were greatly less than the stipulated threshold (5%) by the Food and Drug Administration (FDA), confirming their safety for in vivo application. When blood comes into direct contact with biological materials, protein adsorption and interactions with blood cells may occur, potentially affecting blood compatibility [43,44]. The hydrophilic functional groups within the CPA hydrogels may reduce the interactions with erythrocyte membranes [45], thereby contributing to the observed low hemolysis rates.

Overproduction of ROS can promote oxidative stress, which harms cells and tissues and impedes or delays wound healing. Thus, the intracellular ROS levels of NIH 3T3 cells exposed to CPA hydrogels were evaluated using 2′,7′-dichlorodihydrofluorescein diacetate (DCFH-DA) staining, with H_2_O_2_ used as a ROS stimulator. The fluorescent images are shown in Figure 9d. The H_2_O_2_-treated group exhibited a strong fluorescence signal, indicating increased intracellular ROS accumulation. In contrast, the groups treated with CPA hydrogel extracts exhibited weaker fluorescence signals than the H_2_O_2_-treated group, and the fluorescence intensity appeared close to that of the control group without H_2_O_2_ treatment. These observations suggest that CPA hydrogel may help reduce H_2_O_2_-induced ROS accumulation under the present experimental conditions.

### 3.7. Cytocompatibility Evaluation of CPA Hydrogels

**Figure 10:**
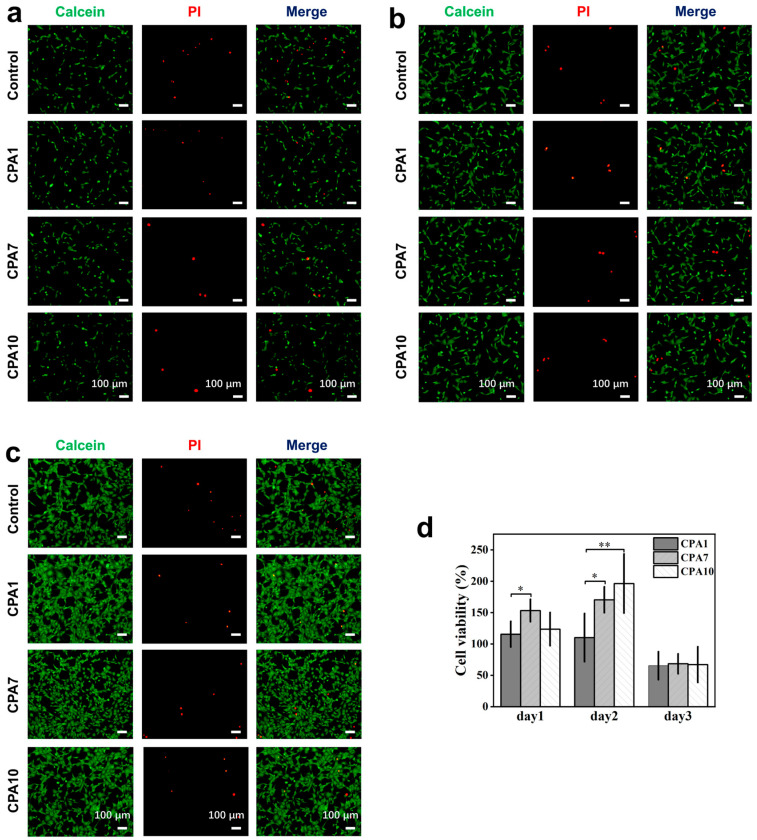
The fluorescent images of NIH 3T3 cells at (**a**) day 1, (**b**) day 2, and (**c**) day 3 in the cytocompatibility test after culturing with CPA1, CPA7, and CPA10 hydrogel extracts. Calcein-AM staining indicates the live cells (green), and PI staining indicates the dead cells (red). (**d**) CCK-8 assay results to measure the cell viability, * *p* < 0.05, and ** *p* < 0.01.

The cytocompatibility of CPA1, CPA7, and CPA10 hydrogels was investigated by culturing the typical NIH 3T3 cells with the corresponding hydrogel extracts. Cell morphology and viability were observed using fluorescence microscopy following Calcein-AM/PI staining (Figure 10a–c), and relative cell viability was further measured by CCK-8 analysis (Figure 10d). A few dead cells (PI staining in red) were observed in all groups, whereas most cells exhibited green Calcein-AM fluorescence. The number of live cells visually increased with culture time. The CCK-8 results were generally consistent with the live/dead staining observation (Figure 10d). CPA7 and CPA10 hydrogels significantly promoted cell proliferation during days 1–2 of culture, with CPA10 showing a highly significant proliferative effect on day 2 (** *p* < 0.01). However, on day 3, cell viability decreased in all treatment groups, which may be related to nutrient depletion, accumulation of metabolic byproducts, or changes in the microenvironment during later stages of culture.

### 3.8. Cell Pro-Migration Ability Evaluation

**Figure 11:**
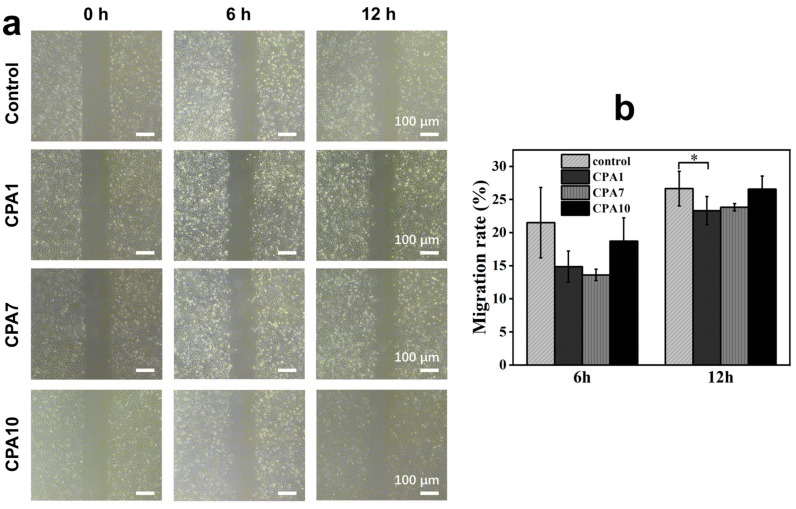
The cell pro-migration abilities facilitated by CPA hydrogels were investigated using the scratch assay. The optical images of NIH 3T3 cells after culturing with (**a**) CPA hydrogel extracts for 6 and 12 h. (**b**) The calculated migration rates of NIH 3T3 cells, * *p* < 0.05.

The effect of the composite hydrogels on cell migration was evaluated using a cell scratch assay. As shown in Figure 11a, the scratch lines were clearly visible at 0 h. After cultivation, only a small number of cells were detected in the scratched area in the hydrogel-treated groups compared to the control group, and the scratch gap remained observable, indicating that the CPA hydrogels did not markedly promote cell migration under the present experimental conditions. Furthermore, the migration rate was quantified using ImageJ, and the results are shown in Figure 11b. The migration rates of most hydrogel-treated groups were lower than those of the control group, although not all differences reached statistical significance. Among them, the CPA10 treatment group showed the migration rate closest to that of the control group. These results suggest that the current CPA hydrogel formulation has a limited ability to directly stimulate cell migration. Therefore, further formulation optimization may be needed to improve the cell-migration response for future tissue-repair applications.

## 4. Conclusions

In summary, 4-CPBA-PEI derivatives were synthesized via EDC/NHS-mediated amidation using PEI with different molecular weights. ^1^H NMR and FT-IR results confirmed the successful formation of amide bonds and the efficient grafting of phenylboronic acid groups onto PEI. The CPA hydrogels were further prepared by mixing 4-CPBA-PEI with PVA. All hydrogels possessed interconnected porous networks, and their pore structure and swelling capacity were effectively regulated by PEI molecular weight. Rheological tests demonstrated typical viscoelasticity, shear-thinning behavior, and excellent self-healing performance. Benefiting from the functional groups in the polymer matrix, the hydrogels exhibited wet-surface attachment capability to various materials and ex vivo biological tissues. Moreover, the CPA hydrogels showed high ABTS radical scavenging activity, good hemocompatibility, and a reduced H_2_O_2_-induced ROS fluorescence signal in NIH 3T3 cells. Cytological tests on NIH 3T3 fibroblasts proved their low cytotoxicity and favorable biocompatibility. In conclusion, the as-prepared hydrogels integrate tunable physical properties, wet-surface attachment, self-healing capability, antioxidant activity, and excellent biocompatibility, suggesting their potential as tissue-contacting wound dressing materials. A limitation of the present study is that the long-term in vitro degradation behavior of the CPA hydrogels was not evaluated. Future work should include systematic mass loss studies in physiological media over 14–21 days to better understand their stability and degradation profile for wound dressing applications.

## Data Availability

Data will be made available on request.
